# Occupational and lifestyle factors associated with SCORE-2 cardiovascular risk among emergency healthcare workers

**DOI:** 10.1007/s11845-026-04429-5

**Published:** 2026-05-05

**Authors:** Murat Doğan, Anıl Özüdoğru

**Affiliations:** 1 Faculty of Medicine, Department of Family Medicine, Kırşehir Ahi Evran University, Kırşehir, Türkiye; 2 School of Physical Therapy and Rehabilitation, Kırşehir Ahi Evran University, Kırşehir, Türkiye

**Keywords:** Cardiovascular risk, SCORE-2, Emergency healthcare workers

## Abstract

**Objectives:**

This study aimed to examine the associations between occupational characteristics, lifestyle behaviors, sleep quality, and 10-year cardiovascular risk estimated using SCORE-2 algorithm among ambulance emergency medical services (EMS) and emergency department healthcare workers.

**Methods:**

This cross-sectional study included 300 healthcare workers employed in ambulance EMS (*n* = 122) and emergency departments (*n* = 178). Sociodemographic variables, occupational characteristics, lifestyle behaviors, and clinical data were collected using a structured questionnaire. Sleep quality was assessed with the Pittsburgh Sleep Quality Index (PSQI), and cardiovascular risk was estimated using SCORE-2. Group comparisons were performed using the Mann–Whitney U and chi-square tests. Associations were evaluated with Spearman correlation, and independent predictors of SCORE-2 were identified using multivariable linear regression analysis.

**Results:**

The median SCORE-2 value was 2.8% (IQR: 1.6–4.7), with no significant difference between ambulance EMS and emergency department workers (*p* = 0.855). Participants aged ≥ 50 years had significantly higher SCORE-2 values compared with those aged 40–49 years (*p* < 0.001). Alcohol consumption was associated with higher SCORE-2 levels (*p* < 0.001) and remained an independent predictor in multivariable analysis (B = 1.41, 95% CI: 0.60–2.22). Age and sex were also independently associated with SCORE-2. Although sleep quality showed a weak positive correlation with SCORE-2 (*r* = 0.166, *p* = 0.004), this association was not significant after adjustment.

**Conclusion:**

In emergency healthcare workers, SCORE-2-estimated cardiovascular risk is mainly driven by age, sex, and alcohol consumption rather than work setting or shift characteristics. These findings suggest that incorporating occupational and lifestyle factors alongside SCORE-2 may improve cardiovascular risk assessment in shift-working healthcare professionals.

## Introduction

Cardiovascular diseases (CVDs) remain among the leading causes of mortality worldwide and represent a major public health concern, particularly among working-age adults [1]. A similar pattern is observed in Türkiye, where cardiovascular diseases account for 37% of all deaths related to non-communicable diseases, ranking first among causes of mortality [2]. Therefore, the early identification of individuals at increased cardiovascular risk is essential for planning effective preventive and intervention strategies, with primary care physicians playing a pivotal role in the initial detection and management of cardiovascular risk.

The Systematic Coronary Risk Evaluation (SCORE) model was originally developed to estimate the 10-year risk of fatal cardiovascular disease in European populations without established atherosclerotic disease; however, it has recently been updated to SCORE2 using contemporary European cohort data [3, 4]. SCORE2 is currently recommended by the European Society of Cardiology (ESC) guidelines for cardiovascular risk assessment in individuals aged 40–69 years without diabetes or established cardiovascular disease [5].

Despite its widespread use, SCORE2 is based solely on traditional risk factors such as age, sex, smoking status, systolic blood pressure, and lipid profile [3, 5]. Consequently, it does not directly account for occupational factors, lifestyle behaviors, or sleep-related characteristics that may influence cardiovascular risk through cumulative or indirect mechanisms. These limitations are particularly relevant for healthcare professionals working in emergency medical settings.

Emergency healthcare workers are frequently exposed to irregular working hours, prolonged 24-hour shifts, high occupational stress, and disrupted sleep patterns, all of which have been associated with adverse cardiometabolic outcomes [6–8]. In addition, lifestyle behaviors such as alcohol consumption, physical inactivity, and poor sleep quality are relatively common in this population and may further increase cardiovascular disease risk [9, 10].

Taken together, data examining how occupational and lifestyle factors relate to SCORE2-derived cardiovascular risk among emergency healthcare workers remain limited. In particular, it remains unclear whether work schedules, sleep quality, and modifiable lifestyle behaviors are associated with predicted cardiovascular risk independently of traditional SCORE2 components.

Despite the widespread use of SCORE-2 in cardiovascular risk assessment, its applicability in high-stress and shift-working populations remains insufficiently explored. Occupational stressors, irregular working hours, and sleep disturbances are not directly incorporated into the SCORE-2 algorithm [11]. This represents an important gap in the literature, especially for emergency healthcare workers who are routinely exposed to such risk factors [12]. Therefore, this study aims to address this gap by evaluating whether occupational and lifestyle-related factors contribute to SCORE-2-estimated cardiovascular risk beyond traditional risk determinants. Therefore, the present study aimed to evaluate the relationship between occupational characteristics, lifestyle factors, sleep quality, and cardiovascular risk estimated using SCORE2 among healthcare workers employed in ambulance emergency medical services (EMS) and emergency departments in Türkiye.

In addition, healthcare workers constitute a unique occupational group in whom conventional cardiovascular risk prediction models may not fully reflect real-life risk profiles. Emergency healthcare professionals are exposed not only to traditional cardiovascular risk factors but also to cumulative occupational stress, circadian rhythm disruption, and lifestyle constraints imposed by irregular working schedules. While tools such as SCORE-2 are widely recommended for population-based risk stratification, their applicability in high-stress, shift-working populations remains insufficiently explored. Evaluating SCORE-2 in the context of occupational and lifestyle characteristics may therefore provide valuable insight into the strengths and limitations of this algorithm in emergency healthcare settings and help identify areas where complementary risk assessment approaches are warranted.

## Methods

### Study design and ethical approval

This cross-sectional study was conducted among healthcare workers employed in ambulance emergency medical services (EMS) and emergency departments in Kırşehir, Türkiye. Ethical approval for the study was obtained from the local ethics committee (Approval No: 148 − 22/10/2024). All procedures were carried out in accordance with the principles of the Declaration of Helsinki. Written informed consent was obtained from all participants prior to enrollment.

### Participants and data collection

Healthcare workers aged between 40 and 69 years, actively working in ambulance EMS or emergency departments, and without a prior diagnosis of cardiovascular disease or diabetes were included in the study. Individuals with incomplete data, known cardiovascular disease, or outside the SCORE-2 target age range were excluded. Participants were recruited using a consecutive sampling approach among eligible individuals who agreed to participate during the study period. After reading and approving the informed consent form, participants who met the inclusion criteria were included in the study. Data were collected using a structured questionnaire administered by the researchers. Sociodemographic characteristics (age, sex, marital status), occupational characteristics (work setting and work schedule), lifestyle behaviors (smoking status, alcohol consumption, regular exercise), and clinical measurements were recorded. Data collection was conducted between January-August 2025. All questionnaires were administered face-to-face by the researcher (DU) during working hours in the respective healthcare settings. Each participant completed the questionnaire under supervision to ensure completeness and accuracy of responses.

### Sample size consideration

The study population consisted of healthcare workers employed in ambulance emergency medical services and emergency departments during the study period. All eligible participants who met the inclusion criteria and agreed to participate were consecutively recruited.

Considering the primary outcome of the study (SCORE-2) and the planned comparative and multivariable regression analyses, the sample size was deemed sufficient to ensure adequate statistical power. Methodological recommendations suggest that multivariable regression models require a minimum of 10–15 participants per independent variable included in the model [13]. In the present study, the final regression model included five independent variables, indicating a minimum required sample size of 75 participants. The final sample size of 300 healthcare workers substantially exceeded this threshold, allowing for robust statistical analyses.

### Clinical measurements and cardiovascular risk evaluation using SCORE-2

Anthropometric measurements, including body mass index (BMI), were calculated using standard methods based on measured height and weight. Systolic blood pressure (SBP), total cholesterol, and high-density lipoprotein (HDL) cholesterol levels were obtained from medical records and/or on-site clinical measurements performed during routine health assessments.

The 10-year risk of cardiovascular disease (CVD) was estimated using the Systematic Coronary Risk Evaluation 2 (SCORE-2) algorithm, which is recommended for European populations aged 40–69 years without established cardiovascular disease or diabetes. The SCORE-2 calculation incorporates age, sex, smoking status, systolic blood pressure, total cholesterol, and HDL cholesterol.

Risk estimation was performed according to the appropriate European risk region classification. In line with current ESC recommendations, SCORE-2 values were calculated as continuous percentage values and used as a continuous variable in statistical analyses. Higher SCORE-2 values indicate a greater estimated 10-year risk of fatal and non-fatal cardiovascular events [4]. 

### Assessment of sleep quality

Sleep quality was assessed using the Pittsburgh Sleep Quality Index (PSQI). The PSQI is a validated instrument that allows sleep quality to be evaluated both qualitatively and quantitatively. The questionnaire consists of a total of 24 items, of which 19 are self-rated by the participant and included in the scoring, while the remaining five items are completed by a roommate or bed partner and are not included in the total score.

The PSQI evaluates seven components: subjective sleep quality, sleep latency, sleep duration, habitual sleep efficiency, sleep disturbances, use of sleeping medication, and daytime dysfunction. The total PSQI score ranges from 0 to 21, with higher scores indicating poorer sleep quality. A total score of 5 or below is considered indicative of good sleep quality, whereas scores above 5 reflect poor sleep quality [14, 15]. 

### Statistical analysis

Statistical analyses were performed using SPSS version 29.0 (IBM Corp., Armonk, NY, USA). Continuous variables were tested for normality using the Kolmogorov–Smirnov and Shapiro–Wilk tests. Because most continuous variables were not normally distributed, they were expressed as median and interquartile range (IQR), while categorical variables were presented as number and percentage. The choice between parametric and non-parametric tests was based on the distribution of the data. Since most continuous variables did not meet normality assumptions, non-parametric tests were preferred for group comparisons and correlation analyses.

Comparisons between two independent groups were performed using the Mann–Whitney U test for continuous variables and the chi-square test for categorical variables. Associations between SCORE-2 and continuous variables (PSQI total score and BMI) were assessed using Spearman’s rank correlation analysis.

To identify independent factors associated with SCORE-2, a multivariable linear regression analysis was conducted. Variables entered into the model included age, sex, alcohol consumption, sleep quality (PSQI total score), and work schedule. Multicollinearity was evaluated using variance inflation factor (VIF) values. A p-value of < 0.05 was considered statistically significant.

## Results

A total of 300 healthcare workers were included in the study, of whom 122 were ambulance EMS personnel and 178 were emergency department staff. The demographic, clinical, and lifestyle characteristics of the participants are presented in Table 1.

**Table 1:** Demographic, clinical, and lifestyle characteristics of participants according to work setting

Variable	All participants (*n* = 300)	Ambulance EMS (*n* = 122)	Emergency Department (*n* = 178)	*p*-value
Demographic characteristics				
Age, years	46.0 (42.0–51.0)	46.5 (41.8–51.3)	46.0 (42.0–51.0)	0.934
Male sex, n (%)	123 (41.0)	55 (45.1)	68 (38.2)	0.234
Married, n (%)	209 (69.7)	86 (70.5)	123 (69.1)	0.797
Anthropometric and clinical variables				
Body mass index, kg/m²	25.0 (22.9–27.8)	25.3 (23.3–28.4)	24.8 (22.4–27.2)	0.169
Systolic blood pressure, mmHg	124 (118–132)	124 (116–135)	125 (120–129)	0.878
Total cholesterol, mg/dL	195 (170–225)	186.6 (161.9–216.1)	202.2 (175.1–231.9)	**0.007**
HDL cholesterol, mg/dL	46 (39–53)	45 (39–52)	47 (39–55)	0.453
SCORE-2, %	2.8 (1.6–4.7)	3.0 (1.5–4.9)	2.6 (1.7–4.5)	0.855
Lifestyle behaviors				
Current smoking, n (%)	101 (33.7)	43 (35.2)	58 (32.6)	0.632
Alcohol consumption, n (%)	27 (9.0)	8 (7.1%)	19 (10.7%)	0.221
Regular exercise, n (%)	76 (25.3)	23 (18.9%)	53 (29.8%)	**0.033**
Occupational characteristics				
8-hour shift work, n (%)	95 (31.7)	9 (7.4)	86 (48.3)	**< 0.001**
24-hour shift work, n (%)	205 (68.3)	113 (92.6)	92 (51.7)	**< 0.001**

*The ‘All participants’ column is provided for descriptive purposes only; p-values represent comparisons between Ambulance EMS and Emergency Department groups.Continuous variables are presented as median (interquartile range) due to non-normal distribution. Categorical variables are presented as number (percentage). Between-group comparisons were performed using the Mann–Whitney U test for continuous variables and the chi-square test for categorical variables. Only systolic blood pressure values were included in the analyses in accordance with the SCORE-2 algorithm.*

In univariate analyses, SCORE-2 values did not differ significantly according to work schedule, regular exercise status, comorbidity, or marital status. However, participants who reported alcohol use had significantly higher SCORE-2 values compared with non-users. No significant differences were observed in SCORE-2 according to smoking status or regular exercise (Table 2).

**Table 2:** Associations of SCORE-2 with occupational and lifestyle factors

Variable	Category / Statistic	SCORE-2, median (IQR)	*p*-value
Age group	40–49 years (*n* = 204)	2.0 (1.0–2.8)	**< 0.001**
	≥ 50 years (*n* = 96)	4.7 (2.6–7.4)	
Work schedule	8-hour shift	3.1 (1.7–4.9)	0.255
	24-hour shift	2.6 (1.5–4.6)	
Regular exercise	Yes	3.45 (1.6–4.75)	0.288
	No	2.60 (1.6–4.6)	
Comorbidity	Yes	2.6 (1.7–4.48)	0.927
	No	2.9 (1.6–4.63)	
Alcohol use	Yes	4.7 (2.6–6.15)	**< 0.001**
	No	2.6 (1.5–4.4)	
PSQI total score (PUKI)	Spearman *r* = 0.166	-	**0.004**
BMI	Spearman *r* = 0.057	-	0.326

*SCORE-2 values are presented as median (interquartile range). Between-group comparisons were performed using the Mann–Whitney U test. Correlations between SCORE-2 and continuous variables were assessed using Spearman’s rank correlation coefficient.*

Correlation analyses revealed a weak but statistically significant positive correlation between SCORE-2 and PSQI total score, indicating higher cardiovascular risk with poorer sleep quality. No significant correlation was found between SCORE-2 and body mass index (BMI) (Table 2).

In multivariable linear regression analysis, age, male sex, and alcohol use emerged as independent predictors of higher SCORE-2 values, whereas sleep quality and work schedule were not independently associated with SCORE-2 after adjustment. The results of the multivariable regression analysis are summarized in Table 3.

**Table 3:** Multivariable linear regression analysis for factors associated with SCORE-2

Variable	B (95% CI)	*p*-value
Age (years)	0.245 (0.206–0.284)	**< 0.001**
Male sex	−1.44 (− 1.91 to − 0.97)	**< 0.001**
Alcohol use (yes)	1.41 (0.60–2.22)	**< 0.001**
PSQI total score	0.03 (− 0.04 to 0.09)	0.408
24-hour shift work	0.08 (− 0.41 to 0.57)	0.747

*Multivariable linear regression analysis was performed with SCORE-2 as the dependent variable. The model was adjusted for age*,* sex*,* alcohol consumption*,* sleep quality (PSQI total score)*,* and work schedule. Regression coefficients (B) are presented with 95% confidence intervals (CI). No significant multicollinearity was detected (all variance inflation factor values < 2).*

A total of 300 healthcare workers were included in the study, comprising 122 ambulance EMS personnel and 178 emergency department workers. The median age of participants was 46.0 years (IQR: 42.0–51.0), with no significant age difference between work settings (*p* = 0.934). Sex distribution and marital status were similar between the two groups.

Among anthropometric and clinical variables, total cholesterol levels were significantly higher in emergency department workers compared with ambulance EMS personnel (*p* = 0.007), whereas body mass index, systolic blood pressure, and HDL cholesterol levels did not differ significantly.

Predicted 10-year cardiovascular risk assessed by SCORE-2 was comparable between ambulance EMS personnel and emergency department workers (*p* = 0.855).

Regarding lifestyle behaviors, regular exercise was more common among emergency department workers (*p* = 0.033), while smoking and alcohol consumption rates did not differ between groups.

SCORE-2 values did not differ according to work schedule (24-hour shift vs. 8-hour shift), regular exercise status, or comorbidity presence (all *p* > 0.05).

Participants who reported alcohol consumption had significantly higher SCORE-2 values compared with non-drinkers (*p* < 0.001).

SCORE-2 showed a weak but significant positive correlation with PSQI total score (Spearman’s *r* = 0.166, *p* = 0.004), whereas no significant correlation was observed between SCORE-2 and body mass index (*r* = 0.057, *p* = 0.326).

In the multivariable linear regression analysis, increasing age was independently associated with higher SCORE-2 values (B = 0.245, 95% CI: 0.206–0.284; *p* < 0.001). Male sex was associated with lower SCORE-2 values compared with female sex (B = − 1.44, 95% CI: −1.91 to − 0.97; *p* < 0.001).

Alcohol consumption remained an independent predictor of higher SCORE-2 scores after adjustment for age, sex, sleep quality, and work schedule (B = 1.41, 95% CI: 0.60–2.22; *p* < 0.001).

In contrast, PSQI total score and 24-hour shift work were not independently associated with SCORE-2 in the fully adjusted model (*p* > 0.05 for both).

## Discussion

The principal finding of this study is that, although no significant difference was observed in overall SCORE-2 levels between ambulance EMS and emergency department workers, age, sex, and alcohol consumption exerted clear and independent effects on SCORE-2. In addition, sleep quality showed a weak but statistically significant association with SCORE-2; however, this relationship lost its independence in multivariable analyses.

SCORE-2 was updated by the European Society of Cardiology in 2021, with European countries reclassified into four distinct risk regions based on contemporary cardiovascular mortality data [4, 5]. Türkiye is categorized among high-risk countries in this classification, suggesting a relatively elevated baseline cardiovascular risk in the general population [5, 16]. In our study, analyses stratified by age group demonstrated significantly higher SCORE-2 values among workers aged 50 years and older, which is consistent with the age-sensitive structure of the SCORE-2 algorithm. Indeed, age represents one of the most influential parameters in SCORE-2 risk stratification.

The protective effects of regular physical activity on cardiovascular risk factors have been well established in the literature [17–19]. Despite the markedly higher prevalence of 24-hour shift work among ambulance EMS personnel, SCORE-2 levels were comparable between ambulance EMS and emergency department workers. Conversely, emergency department workers exhibited higher total cholesterol levels and a greater frequency of regular exercise. These findings suggest that occupational workload and shift patterns may influence cardiovascular risk through indirect pathways. Notably, the SCORE-2 algorithm does not directly incorporate occupational stress, shift work, or physical inactivity [4, 5, 16]. 

Population-based studies have consistently shown that short sleep duration and poor sleep quality are associated with increased risks of cardiovascular disease and coronary heart disease [20–22]. In the present study, PSQI total score demonstrated a weak but positive correlation with SCORE-2; however, this association was no longer significant after adjustment for strong determinants such as age and sex. This finding implies that the cardiovascular effects of impaired sleep quality may be mediated through indirect mechanisms and are only partially captured by the SCORE-2 framework. The lack of an independent association between sleep quality and SCORE-2 in the multivariable model may be explained by the structure of the SCORE-2 algorithm, which primarily incorporates traditional cardiovascular risk factors. In shift-working populations, sleep disturbances may contribute to cardiovascular risk indirectly through mechanisms such as hypertension, metabolic dysregulation, and inflammation [23]. Therefore, the impact of sleep quality may not be fully captured by SCORE-2, which could explain the discrepancy between our findings and previous literature on shift workers.

The association between shift work, long working hours, and cardiovascular risk has been widely reported. Virtanen et al. demonstrated that prolonged working hours and occupational stress increase the risk of cardiovascular events [24]. Similarly, Wong et al. reported a more unfavorable metabolic and cardiovascular risk profile among individuals engaged in shift and night work [25]. In contrast, the absence of an independent association between 24-hour shift work and SCORE-2 in our study suggests that the cardiovascular consequences of shift work may emerge through long-term pathways that are not fully reflected within the SCORE-2 algorithm.

One of the most notable findings of this study is the strong association between alcohol consumption and SCORE-2 observed in univariable analyses, which persisted as an independent predictor in multivariable models. The continued independent effect of alcohol use on SCORE-2 aligns with existing evidence indicating that alcohol consumption can increase cardiovascular risk through adverse effects on lipid profiles, blood pressure, and insulin resistance [26, 27]. Moreover, alcohol intake has been shown to be positively associated with blood pressure levels and hypertension prevalence, to increase the risk of stroke at higher consumption levels, and to reduce the effectiveness of antihypertensive treatment [28–30]. In this context, the identification of alcohol use as a significant determinant of SCORE-2 among emergency healthcare workers is clinically relevant and highlights a potentially modifiable risk factor.

The strengths of this study include the evaluation of SCORE-2 alongside occupational and lifestyle factors within a unique and high-stress professional group of emergency healthcare workers. Additionally, the use of both univariable and multivariable analyses enhances the robustness and interpretability of the findings. Nevertheless, several limitations should be acknowledged. Due to the cross-sectional design, causal relationships cannot be established, and the association between SCORE-2 and actual cardiovascular events could not be assessed. Furthermore, certain occupation-related factors, such as psychosocial stress, were not directly measured. In addition, lifestyle-related variables were based on self-reported data, which may be subject to recall or reporting bias. The study was conducted in a single geographical region, which may limit the generalizability of the findings to other populations. Furthermore, the cross-sectional design precludes causal inferences between the studied variables and cardiovascular risk.

The findings of the present study underscore the importance of modifiable lifestyle factors in shaping cardiovascular risk among emergency healthcare workers. Although occupational variables such as shift type and work setting were not independently associated with SCORE-2, alcohol consumption emerged as a consistent and independent determinant of increased cardiovascular risk. This observation suggests that lifestyle behaviors may exert a more direct influence on SCORE-2 components than occupational characteristics per se. From a preventive perspective, these results highlight the potential value of workplace-based health promotion strategies focusing on alcohol use reduction and lifestyle counseling, particularly in emergency healthcare environments where behavioral risk factors may be amplified by occupational stress and time constraints. Incorporating such interventions alongside routine cardiovascular risk screening may enhance early prevention efforts in this vulnerable workforce.

From an occupational health perspective, these findings highlight the importance of integrating cardiovascular risk screening into routine health assessments for emergency healthcare workers. In addition, workplace-based interventions targeting modifiable lifestyle factors, such as alcohol consumption, sleep hygiene, and physical activity, may contribute to reducing long-term cardiovascular risk. Organizational strategies, including optimization of shift schedules and workload management, may also support cardiovascular health in this population.

In conclusion, our findings suggest that while SCORE-2 serves as a useful indicator of overall cardiovascular risk among emergency healthcare workers, it may inadequately capture the contributions of alcohol use, sleep quality, and occupational burden. Therefore, particularly in shift-working healthcare personnel, SCORE-2 may be more informative when complemented by additional assessments that incorporate lifestyle behaviors and occupational risk factors. Future prospective studies are warranted to support the development of expanded risk models tailored to this specific population. In practical terms, periodic cardiovascular risk screening using tools such as SCORE-2 may be particularly beneficial in emergency healthcare workers. Additionally, interventions focusing on sleep hygiene education, promotion of healthy lifestyle behaviors, and optimization of shift scheduling should be considered as part of comprehensive occupational health programs.
